# Supplementary material for: Updated estimation of cutaneous leishmaniasis incubation period in French Guiana
Source: PLoS Negl Trop Dis. 2023 Jun 14;17(6):e0011415. doi: 10.1371/journal.pntd.0011415 (PMC10298763; doi:10.1371/journal.pntd.0011415)
Supplement: Attachment — Submitted filename: Answers to reviewers - PNTD-08 02 2023_SB.docx [file pntd.0011415.s007.docx]

**Manuscript -** Ref. No.: PNTD-D-22-01015

**Title:** Cutaneous Leishmaniasis incubation period in French Guiana: a paradigm shift

**Corresponding author:**

Dr Romain Blaizot (blaizot.romain@gmail.com), Cayenne Hospital Center, Dermatology Department, Cayenne, French Guiana.

Dear Editorial-Board Member,

According to Editorial-board and Reviewer comments, revisions to the edited version of our manuscript headed “**Cutaneous Leishmaniasis incubation period in French Guiana: a paradigm shift**” have been performed. We hope that our revisions to the originally submitted version of the manuscript will correspond to the Editorial-board and reviewer expectations and are in compliance with the *PLOS Neglected Tropical Diseases* guidelines. Changes were included in our manuscript with track changes as a word document and are also indicated in the cover letter (see below). A definitive version without track changes is also provided as requested.

Sincerely Yours,

Romain Blaizot

General comments addressed to the editor and reviewers relating to the major modifications that have been performed in the manuscript.

We thank the reviewers for their insightful comments that lead to major modifications in our manuscript.

**Reviewer #1**

***Major concerns***

**1)**	**Very interesting study addressing a poorly described parameter of leishmaniasis' epidemiology. However, the data presented needs to be properly expanded to better understand what conclusions can be obtained from it. Also, the limited nature of the surveillance record data available prevents from making solid conclusions. A paradigm shift would not result only from this preliminary data, but would require a properly conducted validation study. And in general, more specific acknowledgement of limitations is needed, and their impact in the strength of the conclusions, and the actual possibility of a “paradigm shift”.**

**Response:** Here, we assessed cutaneous leishmaniasis (CL) incubation period (IP) distribution indirectly, using dates of travel and symptom onsets from military personnels that travelled from non-endemic areas to French Guiana for field training. Importantly, a direct monitoring of IP would not be possible because the date of exposure is often not known for vector borne diseases. To our knowledge, estimating IP distribution with interval censored analysis of travel and symptom onset dates is the best available ethical alternative. We screened for all reported CL cases over a period of 20 years in this region and we estimated CL IP distribution based on a subset of 180 cases out of 626 unique cases, after data filtering. We agree that the “paradigm shift” term could be overstated and we proposed an alternative title: “Updated estimation of Cutaneous Leishmaniasis incubation period in French Guiana”. We inferred IP probabilities with confidence intervals over time after infection using dedicated tools (time-to-event models) that are adapted to interval censoring (they deal with the time of infection uncertainty inside a time interval). To our knowledge, this work is the first to implement time-to-event strategies to estimate CL IP distribution using an unprecedented number of cases. The manuscript is now updated to better describe data collection and screening. A PRISMA related flow diagram that summarizes the procedure is also now provided. Importantly, we now give access to all data and analysis scripts that we used in this work in Supplementary files.

**2)**	**There is insufficient description of the nature and contents of the surveillance records used, how they are collected, where data was recorded (in France, in French Guiana or both), how records were extracted for the study, etc. A standard PRISMA flowchart should accompany the manuscript to understand how much data was eliminated in order to reach the study sample. Also, how in the records it was determined that personnel were in leishmania endemic areas? A deployment in Cayenne city would count as being in endemic areas ? How was this accounted for in the existing records? More description on what was done (and not done) is needed. How does this affect the accuracy of the incubation period estimates?**

**Response:** We thank the reviewer for this suggestion. We added a sentence in Methods to provide more description of the data: “*All information about CL cases was collected during 2001–2021, in France or in French Guiana, by the military physicians by using a mandatory, specific form (C4). This form includes socio-demographic information (age, gender, military unit), clinical signs (symptoms onset date, lesions number, localization, dimension and dissemination) and outcomes (cure or failure), diagnostic tests (smear, culture on skin biopsy and PCR on skin biopsy or swab), treatment data (type, date, mode of administration and side effects) and travel data (location, arrival and departure dates)*”. A PRISMA flow chart is now provided in Supplementary Fig S4. The reviewer raised an interesting question relative to how the length of the stay period (or the accuracy at determining it) can influence the IP distribution estimation with our method. While not being specialists of time to event statistics applied to interval censored data, but rather users of available methods developed by others, we can assume that lengths of stay durations in areas of possible exposure would impact the distribution of all possible times where NPMLE (the maximum probability of IP) can change (called Tunbull intervals), producing higher densities of these intervals over a given time range. IP distribution estimation would then be more accurate from a set of short periods than long periods, because it would thereby approximate exactly observed data. The strategy to select only for short exposure periods was implemented by others to assess IP distributions for vector-borne pathogens. Here, the periods of probable infection were limited to areas with a known exposure potential to phlebotomine sand flies and *Leishmania* transmission, that is to say: (i) training fields such as the “Centre d'entraînement en forêt équatoriale (CEFE)” or (ii) deep forest during operations combating illegal gold mining activities. Therefore, all exposition periods are informative of the IP distribution, independently of their duration. The only general assumption that can restrict the use of censored data analysis is that censoring times and event times are independent. Our method implies this assumption. To summarize this, short exposition periods would be more informative than long exposition periods to estimate IP distribution, but all of them can inform the model. We reckon that our IP distribution estimate is the best proxy of the true underlying IP distribution that we can make given our data and limitations.

**3)**	**There is insufficient description of the actual results. No data is presented on the minimum and maximum incubation periods, nor the minimum or maximum travel period. No table with descriptive data is presented. Greater emphasis is placed in estimates from statistical calculations that in minimally descriptive data (how about an histogram of actual observed incubation periods?). This is at odds with statements about how long can the incubation period be, because no data is provided as reference in this case series. Apparently there were several cases that had incubations periods > 2 months, but it’s hard to understand that in the paper itself.**

**Response:** Interval censoring occurs when a response is known to occur only in an interval. In our case, it is not possible to know the date of pathogen exposure, but it is possible to delimit a period in which the infectious bites have occurred for each individual. We do not have the option of examining histograms for interval censored data as we would if the outcome was uncensored. We are using travel and symptom onset dates as raw data and we infer the incubation period distribution using adapted statistical methods. Please refer to the work of Miranda Chan and Michael A. Johansson, 2012 or to the IcenReg R package vignette to have more method details ^1^ ^2^.

Reference data about CL are scarce. Aoun and colleagues estimated a median IP of 35 days with a range from 7 to 147 days in Tunisia using 92 cases. These 92 samples resulted from a very strict data filtering to select trips in endemic areas shorter than a week to thus infer IP with an accuracy of one week, allowing the authors to estimate an IP distribution by considering IP as directly observed continuous data and by thereby considering the uncertainty of the time to observation as null (no censoring). Oré and colleagues estimated a median IP of 56 days with min 14 days and max 252 days in the Amazon basin using a similar method. This is one way to do it. Another way is to use time to event models that are fully adapted to analyze the time until an event occurs when time is measured at discrete intervals and in the presence of censoring. In time-to-event models (or survival analysis), the main outcome is the likelihood of the occurrence of a specific event over time, and the research question is how this likelihood is impacted by predictor variables. We can easily infer probabilities of observing the event at a given time with confidence intervals using these models. In addition, we did not have to select only for short exposition periods, or to bin event probabilities within a short time window, all exposure periods are informative for these models, independently of their duration. Supplementary Fig S5 is describing raw data with arrival, departure and symptom onset dates for each individual. Raw data are now provided in Supplementary file S2 as a tabulated file. This file can be directly loaded in the R analysis script now provided as Supplementary file S1. The reviewer is right concerning the presence of long incubation periods; they indeed exceed 62.15 days (95% CI, 56–69.5 days) in 5% of cases as described in the result section and Fig 2. We rewrote some sections of the manuscript to try to gain clarity.

Table 1 also provides new descriptive data on the clinical symptoms and epidemiology of the study population.

**4)**	**An important emphasis is placed in statistical methods, but the choices are not well justified (why NPMLEs? Why AFT regression over proportional hazards?). Some answers may be somewhat obvious but others are not. Two estimates are presented sometimes, which is the right one and why is the other presented? Again, no tables are presented with regression coefficients, confidence/credible intervals, etc. This affects the readability of the manuscript.**

**Response:** Three different types of models can indeed be used in survival analysis: non-parametric method (Kaplan-Maier estimates), Cox proportional hazard model (semi-parametric) and accelerated failure time model (AFT, parametric). Non-Parametric Maximum Likelihood Estimator (NPMLE), also referred to as Turnbull’s Estimator, is a generalization of the Kaplan Meier curves that allows for interval censoring. Unlike the Kaplan Meier curves, the solution is not in closed form (refer to response 3). Non-parametric analysis is not constrained by the assumption of a constant hazard ratio over time or a multiplicative relationship between the predictors and the hazard, unlike Cox proportional hazard model. NPMLE is thus the most flexible method but, when using interval censored data, can only give survival estimates as time intervals (i.e. the probability of occurrence of an event is the same over the interval). The parametric method (AFT) has the asset to provide a smooth survival function and to constrain the model flexibility, which thereby give more accurate estimation under the assumption that the chosen survival function distribution fits the data. The choices of parametric baseline distributions in interval censored data can only be done with visual diagnostics by plotting several choices of parametric baseline distributions against the semi-parametric estimates. We have chosen the gamma distribution that superimposes well with the Kaplan-Maier estimates. Supplementary Figure S3-A displays the fit of the gamma baseline model to our data. Importantly, the choice of the baseline (eg. Loglogistic, gamma, lnorm) models do not impact much incubation period estimates (i.e. less than one day shift with the data fit to a gamma or loglogistic distributions, this can be easily assessed using the R script in supplementary file S1). Semi-parametric model would give the same time event distribution as NPMLE in absence of covariates. They would just outperform NPMLE by allowing patients to have different survival functions within the same fitted model whereas the non-parametric approach would fit different models on different subpopulations before comparing them. We preferred to use AFT rather than a semi-parametric model to describe the effect of lesion dissemination because it is easier to interpret and does not require a proportional hazard assumption criterion. AFT parameters were estimated using a Bayesian Accelerated Failure Time model for interval censored data with flat priors. In our conditions (i.e. flat priors), it is giving very similar coefficient estimates than a classic parametric regression model, with the advantage to visualize trace plots of Markov Chain Monte Carlo (MCMC) output and marginal density estimates for each coefficient (Supplementary Fig S3-C). R code to perform classic AFT is provided in Supplementary file S1. A summary table for Bayesian AFT is now provided in Supplementary figure S3-B. Importantly, there are no true or wrong estimates, they both come from different analysis methods. The AFT estimate was however put forward throughout the manuscript to gain clarity. A basic explanation of the asset and drawback of each method is also now provided.

***Supplementary Figure S3: Bayesian Accelerated Failure Time model coefficient estimation with the gamma baseline model describing the distribution of cutaneous Leishmaniasis incubation period.*** *A- Diagnosis plot that displays the fit of the parametric gamma baseline model to Kaplan-Maier estimates. B- Summary table of the Bayesian Accelerated Failure Time model. C- Trace plots of Markov Chain Monte Carlo (MCMC) output and marginal density estimates for each gamma baseline distribution coefficient estimated with a Bayesian Accelerated Failure Time model for interval censored data with flat priors.*

***Result section:*** *“This non-parametric method adapted to interval censoring is not constrained by conditions of application, but only give survival estimates as time intervals (i.e. the probability of occurrence of an event is the same over the interval). Accelerated failure time (AFT) parametric models have the asset to provide a smooth survival function and to constrain the model flexibility, which thereby give more accurate estimation under the assumption that the chosen survival function distribution fits the data.”*

**5)**	**Given that the authors postulate that their observed IP are shorter than previously described in the literature, the immediate question is, how accurate is the surveillance system to detect leish cases with long incubation periods? Could they have been missed, resulting in underestimation of the median/mean IP? Could further deployments of military personal soon after French Guiana prevented the ability of the records to attribute exposure to their stay in French Guiana, missing infections with longer incubations periods? Supplementary Fig S5 shows that the vast majority of leish diagnoses were made before departure, suggesting that diagnoses made back. It is likely that cases with longer incubation periods could have been misdiagnosed back home, biasing estimations. Only a prospective study with long-follow up could properly address this issue.**

**Response:** Supplementary Fig S5 shows that the vast majority of leishmaniasis diagnostics were made after departure and not before. The vertical line represents symptom onsets, and the green points represent departures from French Guiana. Leishmaniasis diagnostics could be traced back to a single exposure period in 92% (166/180) of cases even after prolonged times post departure (> 70 days). We however cannot dismiss the hypothesis that we could have missed some cases. The probability of failure to detect CL might indeed increase as the duration of the departure to symptom onset period increases. We agree with the reviewer that a prospective study with long follow-up would be more adapted. We lost an important number of records simply because the date of travel was not available when using retrospective data. However, prospective studies are not straightforward to implement, especially when dealing with long follow-up for a disease that has a relatively low incidence. We now discuss this limitation in the discussion section.

**6)**	**The authors describe incubations periods in the literature as short as a week, but Supp Figure 1 includes multiple cases with incubation periods shorter than five days, and even shorter. This is biologically very unlikely and suggests a potential problem in the recording of the symptom initiation or the confirmation of diagnoses, casting doubt on the short incubation period estimates. This issue needs to be recognized and address in the limitations of the study.**

**Response:** No case of incubation period shorter than 5 days are shown in Supplementary Fig S5 (refer to response to comment 5). The minimum Arrival - Symptom onset (AS) period is 8 days and the minimum possible IP for individuals experiencing symptoms after departure lies in intervals [1 - 16 days] or [2 - 14 days]. From our experience we can recall several cases of very short incubations (ie < 5 days) when patients presented with disseminated lesions less than a week after exposure. For example, we had the case of a 30-year-old patient who went on a trip in the forest just after landing from mainland France, presented disseminated papules and ulcerations 5 days later, with a positive smear and a complete cure after a dose of pentamidine. However, for the majority of patients seen in French Guiana, as in other endemic countries, the incubation period cannot be determined as exposition periods are either permanent or recurrent. This is why we chose to use only the military population for this study, as there was only a handful of civilian cases where the IP could be precisely determined.

**7)**	**Time trends over 20 years and Leishmaniasis outbreaks, a very common situation in military deployments, can severely impact estimates. Outbreaks result in highly correlated events, violating the independence assumption of the tests used, and potentially altering both point estimates as well as confidence intervals. Time trends, potentially with fewer military deployments and less cases in recent years could lead to estimates based in older and potentially more error-prone data. A thorough analysis of the impact and role of these two factors in the results are needed.**

**Response:** The reviewer made a pertinent comment. We indeed assumed that the probabilities of CL incubation period over time post infection were not influenced by the data collection date. To check this assumption, our dataset was divided in three sections: (i) a first section with infections occurring before 2006, (ii) a second section with infections occurring between 2006 and 2012 and (iii) a third section with all leishmaniasis cases occurring after 2012. The histogram representing leishmaniasis cases over time with a color code assigned to each section is presented in Supplementary Fig S7 with corresponding IP distribution estimates. Probabilities of CL IP over time were not significantly influenced by time of occurrence of the disease. However, we do not think that older data would be better prone to errors than newer ones. We are working here with simple travel and symptom onset dates. Their accuracy is not supposed to be related to the time of collection.

**8)**	**I am not sure it’s accurate to present the proportion of Guyanensis cases as 30/180 (16.7%), as 30/30 samples were positive (100% positivity) and 16.7% only reflects the testing rate, not the positivity rate. However, it would be relevant to know whether more samples were tested and results were inconclusive. And, I imagine results mostly reflect recent years where species differentiation started to be performed, with obvious biases.**

**Response:** We agree with the reviewer on this point. We have modified the corresponding sentences in the Results section : “*The infecting species was isolated in only 31/180 cases (17.2%) and was always L. guyanensis (31/31, 100%)*”.

Indeed, the characterization of species could not be performed for the entire study period and our results reflect only the data corresponding to specific years. This point is addressed in the discussion.

***Minor concerns:***

**9)**	**Regarding the pre-recruitment status of the study participants: were they screened for already existing CL lesions or travel history to CL endemic areas, prior to deployment to French Guiana? You may mention these in the manuscript or mention as limitations.**

**Response:** Indeed, though they do not see a dermatologist before their arrival in FG, all soldiers do benefit from a consultation with a military general practitioner prior to their deployment. Annual GP consultations are also compulsory. Therefore, it is highly unlikely that pre-existing CL lesions would have been missed. The following sentence has been added to the Methods section: **“***Of note, all military personnel deployed to FG benefit from a consultation with a general practitioner on an annual basis and prior to any deployment.*”, lines 142-144.

**10)** **Please mention exclusion criteria when describing the study population.**

**Response:** Exclusion criteria are now fully presented in the PRISMA flow chart. CL cases after travel to a country other than French Guiana were excluded.

**11)** **Regarding the sample size: please clarify whether 180 is the whole study population or whether it was a selected sample. If it is a selected sample, please mention the sample size calculation and the sampling technique.**

**Response:** These 180 patients represented the whole study population. This is now made clear in the Flow chart (Supplementary Fig S4-A). We also added the following words “and constituted the study population” in the Results section.

**12)** **Regarding statistical analysis: please include the justification for using non-parametric approaches for distribution identification and for assessing the independence of variables. It will add clarity, if the reason behind using a Bayesian AFT regression model is included to the manuscript.**

**Response:** Please refer to answer 4. Nonparametric maximum likelihood estimators (NPMLE) are not constrained by the assumption of a constant hazard ratio over time or a multiplicative relationship between the predictors and the hazard. NPMLE is thus the most flexible method but, when using interval censored data, can only give survival estimates as time intervals (i.e. the probability of occurrence of an event is the same over the interval). The parametric method (AFT) has the asset to provide a smooth survival function and to constrain the model flexibility, which thereby give more accurate estimation under the assumption that the chosen survival function distribution fits the data.

**13)** **It is mentioned in the analysis plan that IP distribution identification will be done with parameter estimation. However, result of the proposed distribution identification is not clearly given in the results section.**

**Response:** All parameter estimates (shape and scale parameter of the model with a gamma distribution) are now provided in Supplementary Fig S3. The R code and raw data are also provided in Supplementary files S1 and S2 with command lines that would give the opportunity to the reader to obtain IP estimates with confidence/credibility intervals at any quartile of the distribution.

***Result section:*** *“The shape and scale of the gamma distribution were estimated to 1.075 (mean) ± 0.1197 (SD) and 2.31 ± 0.127, respectively.”*

**14)** **It is mentioned ‘An AFT with a gamma error distribution was fit with a Bayesian regression model to provide Bayesian estimates.’ Interpretation of the regression estimates relating to the application has not been presented in the manuscript.**

**Response:** The parametric method (AFT) provides a smooth survival function and thus more accurate estimations under the assumption that the chosen survival function distribution fits the data. Anyone can use our gamma distribution parameter estimates to predict CL IP at a given probabilities with their corresponding credible intervals. Supplementary file S1 now provides all the functions to do this.

***Supplementary file S1: R code to analyze interval censored time-to-event data and create visualizations.*** *The file provides code lines used to analyze the tabulated data provided in Supplementary file S2. This file takes Supplementary file S2 as an input file.*

**15)** **To add clarity and value to the manuscript, please include the equation of the final fitted AFT regression model.**

**Response:** The gamma distribution is parameterized in terms of a shape parameter and a rate parameter. Both estimates are now provided in log scale in Supplementary Fig S3. Importantly, IP estimates with their credible intervals can be easily inferred from our data using the survCIs(bayes_fit, p=0.5) function with a specified IP probability (here 50%) (Supplementary file S1).

**16) Line 1: Upper case ‘L’ in Leishmaniasis change to lower case. Line 27: Upper case ‘L’ in Leishmaniasis change to lower case. Line 57: Upper case ‘L’ in Leishmaniasis change to lower case. Line 71: Upper case ‘L’ in Leishmaniasis change to lower case. Line 74: sandflies change to sandfly. Line 86: et al needs to be in italics. Line 97: Soldiers change to Soldiers’ . Line 209: Upper case ‘L’ in Leishmaniasis change to lower case. Line 221: Upper case ‘L’ in Leishmaniasis change to lower case.**

**Response:** We thank the reviewer for these observations. All these mistyping have been corrected accordingly.

**Reviewer #2**

***Major concerns***

**21)** **Page 5, lines 80-82. The authors refer to the ancient experiment on the use of live vaccines to induce preventive sores. This information is out of context, because in this condition there is no participation of the saliva of the vector, which has an important role to recruit cells and also facilitate the entry of the parasite into the host cell.**

**Response:** We thank the reviewer for underlining the role of vector saliva, which indeed makes this information irrelevant. Therefore, this sentence has been removed.

**22)** **Page 8, line 124. The authors cited CL skin lesion, however they not mentioned the type and characteristic of the lesion. So I ask: What was the characteristic lesion? This information is important, because the beginning of the LC lesion is a papule, which evolves into a nodule and then ulcerates, so according to the lesion characterized, there may be a difference in the interpretation of the incubation period.**

**Response:** We agree with the reviewer that papules occur at an earlier stage of cutaneous leishmaniasis. However, compatible lesions in our study were wet ulcers with raised borders, typical of CL. In the text, “compatible lesions “ have been replaced with “typical wet ulcers with raised borders”, lines 121.

**23)** **Regards trip to endemic area: Were only patients included who had their first trip to FG? Or were there some patients like more than one trip?**

**Response:** Patients with several trips (n = 120) were removed before selecting the study population of patients (cf. Supplementary Fig S4-A corresponding to the Prisma 2020 Flow chart).

**24)** **Results. According to the authors, 30 from 180 samples were characterized as L. guyanensis. This sampling is too small to say that L. guyanensis is the main species, because in different areas of the FG there may be circulation of other species, such as L. braziliensis. The authors also did not inform how the species was characterized.**

**Response:** We agree with the reviewer. Our intention was not to state that *L. guyanensis* was the main species in the whole military population, though that assumption would be consistent with regular surveillance data, published outbreaks of CL in soldiers in French Guiana and general data in the civilian population where *L. guyanensis* represents more than 80% of cases. We completely agree that this proportion of species identification is very small, as these techniques were not developed before 2014 and often not performed even afterwards. We modified some of our conclusions to take these limitations into account, lines 354-356 “*The impact of the species involved could not be evaluated as it was determined in only 30 soldiers and cases of L. braziliensis might have been missed. Incubation in species other than L. guyanensis could be different. However, the characterization of species was not available throughout the entire study period, which represents a bias*.”

We also added these following information on species characterization, lines 138 -142: “*Species were determined from 2014 to 2019 with a PCR-RFLP technique targeting a 615-bp region of the RNA Pol II gene on skin biopsies, and SYBR Green-based real time PCR targeting Hsp70 and followed by Sanger sequencing on swab samplings, after 2019, according to previously published studies of the Cayenne Hospital Center.*”

**25)** **Regarding the distribution of the patients over the 20 years of studies, some points need to become clearer.**

**Response:** Distribution of cutaneous leishmaniasis cases in our studied population over time is now represented in Supplementary Fig S4 (number of CL cases that have been included in the study out of the total distribution of the total recorded CL cases) and in Supplementary Fig S7 (refer to answer #7).

**26)** **How many patients had the disseminated cutaneous form? Was there a difference between the years of inclusion of the patients? The authors evaluated a period of 20 years. It is important to demonstrate the average number of cases per year.**

**Response:** We thank the reviewer for this very important question. Cases of disseminated CL were recorded only since 2010, so there was indeed a sharp difference in the years of inclusion. These data have been added to the Results sections (lines 215-216).

“Twelve cases of disseminated CL were recorded, including 8 in 2014 and 4 in 2020.”

**27)** **Discussion. The authors suggest that the strains of *L. guyanensis* causing LCL are different from the strains causing LCL but this has not been demonstrated and there is no evidence for this species.**

**Response:** We agree with the reviewer that this has not been demonstrated with *L. guyanensis.* However, one can assume that if genetic factors give specific strains the ability to provoke DCL among *L. braziliensis* cases, a similar mechanism could be expected in *L. guyanensis* infections. However, it is true that clinical and genetic studies should validate this assumption. We added the following sentences in the discussion to clarify this point: “It is possible that similar genetic factors apply to *L. guyanensis* strains causing DCL” lines 340-341.

“Further studies should also look for associations between specific *L. guyanensis* strains and the occurrence of DCL.” Lines 375 -376.

**28)** **Another important point is about the development of lesions in prime-infected people and those residing in transmission areas, which are frequently exposed to the bite of the vector insect. I suggest to include this in the discussion.**

**Response:** We thank the reviewer for this comment and completely agree. The following sentence has been added to the discussion: **“***It is also noteworthy that the IP in prime-infected patients such as these soldiers could be shorter than in people living in endemic areas, as previous CL infections are known to provide an acquired immunity.*”

We hope these changes will meet your expectations and thank you again for considering this manuscript,

Best regards

Dr Romain Blaizot
