# Supplementary material for: Updated estimation of cutaneous leishmaniasis incubation period in French Guiana
Source: PLoS Negl Trop Dis. 2023 Jun 14;17(6):e0011415. doi: 10.1371/journal.pntd.0011415 (PMC10298763; doi:10.1371/journal.pntd.0011415)
Supplement: Attachment — Submitted filename: Answers to reviewers round 2.docx [file pntd.0011415.s008.docx]

Dear Editorial-Board Member,

We thank you and independent reviewers for your reviewing and positive comments. The edited version of our manuscript headed “**Updated estimation of cutaneous leishmaniasis incubation period in French Guiana**” was modified accordingly. Changes were included in our manuscript with track changes as a word document and are also indicated in the cover letter (see below). A definitive version without track changes is also provided as requested. We hope that our revisions will correspond to the Editorial-board and reviewer expectations.

Sincerely Yours,

Romain Blaizot

Comments:

1) **Include the PRISMA flowchart in the manuscript and not in the supplementary material.**

**Response:** The PRISMA flowchart is now included in the manuscript.

2) **I would not consider the term "contamination" to describe exposure to sandfly bytes or exposure to CL.**

**Response:** “Contamination” was replaced by “exposure” or “sandfly bite” throughout the manuscript.

3) **Your responses in the letter, particularly in the justification of methods used and decisions made, are important and informative. They clarify concerns of the reviewers that most lilkely will be concerns of readers as well. Please include them in the actual manuscript, You can summarize them a little bit if felt needed**

**Response:** We thank the reviewer for this positive comment. We include some justification of methods from the letter to the manuscript. We explained the asset of using survival analysis to estimate incubation periods in the discussion section. In addition, we will take the opportunity to make the peer review history publicly available.

Line 293: “Aoun and colleagues estimated a median IP of 35 days with a range from 7 to 147 days in Tunisia using 92 cases. These 92 samples resulted from a very strict data filtering to select trips in endemic areas shorter than a week to infer IP with an accuracy of one week, allowing the authors to estimate an IP distribution by considering IP as directly observed continuous data. Oré and colleagues estimated a median IP of 56 days with min 14 days and max 252 days in the Amazon basin using a similar method.”

Line 301: “Our method considers the uncertainty of the time to observation (censoring) and infers probabilities of observing an event at a given time without the need to select only for short periods of exposure.”

4) **Some descriptions of the methods are presented in the results when describing the findings. They should be moved to the methods.**

**Response:** The text section below that was originally in the results section is now moved to the material and method section.

Line 186: “NPMLE is a generalization of the Kaplan Meier curves that allows for interval censoring. This method is not constrained by conditions of application, but only give survival estimates as time intervals (*i.e*. the probability of occurrence of an event is the same over the interval). Accelerated failure time (AFT) parametric models have the asset to provide a smooth survival function and to constrain the model flexibility, which thereby give more accurate estimation under the assumption that the chosen survival function distribution fits the data.”
